# DREADDs in Epilepsy Research: Network-Based Review

**DOI:** 10.3389/fnmol.2022.863003

**Published:** 2022-04-07

**Authors:** John-Sebastian Mueller, Fabio Cesar Tescarollo, Hai Sun

**Affiliations:** Department of Neurosurgery, Robert Wood Johnson Medical School, New Brunswick, NJ, United States

**Keywords:** DREADD = designer receptor exclusively activated by designer drugs, epileptogenesis, epilepsy, ictogenesis, seizure, chemogenetic, pharmaco-genetic

## Abstract

Epilepsy can be interpreted as altered brain rhythms from overexcitation or insufficient inhibition. Chemogenetic tools have revolutionized neuroscience research because they allow “on demand” excitation or inhibition of neurons with high cellular specificity. Designer Receptors Exclusively Activated by Designer Drugs (DREADDs) are the most frequently used chemogenetic techniques in epilepsy research. These engineered muscarinic receptors allow researchers to excite or inhibit targeted neurons with exogenous ligands. As a result, DREADDs have been applied to investigate the underlying cellular and network mechanisms of epilepsy. Here, we review the existing literature that has applied DREADDs to understand the pathophysiology of epilepsy. The aim of this review is to provide a general introduction to DREADDs with a focus on summarizing the current main findings in experimental epilepsy research using these techniques. Furthermore, we explore how DREADDs may be applied therapeutically as highly innovative treatments for epilepsy.

## Introduction

Epilepsy is a disorder that is generally perceived as an imbalance between excitation and inhibition in the brain. According to the International League Against Epilepsy, this disorder affects approximately 65 million people worldwide (Devinsky et al., 2018). Epilepsy leads to physical, cognitive, psychological, and social impairments (Fisher et al., 2014; Falco-Walter et al., 2018), and is a major risk factor for sudden unexpected death in epilepsy (Saetre and Abdelnoor, 2018). Historically, epilepsy research has yielded significant drug discoveries driven by the fields of biochemistry and pharmacology. These anti-seizure drugs (ASDs) have helped patients by eliminating or reducing seizures. Unfortunately, ASDs have rarely been able to provide the ideal effect of immediately nullifying seizures with minimal adverse effects in all patients (Engel, 2016). Despite the continuous development of ASDs, more than 30% of patients continue to have seizures after attempted treatment with multiple ASDs; a condition defined as drug refractory epilepsy (Kwan and Brodie, 2000; Sheng et al., 2017). When drug refractory epilepsies have a focal onset, surgical resection of the epileptic foci can be efficacious in rendering patients seizure free (Sheng et al., 2017). Other approaches for the treatment of drug refractory epilepsies include neuromodulation such as vagal nerve stimulation (Morris et al., 2013) and the ketogenic diet (Kossoff et al., 2018). There is a continuing need to develop new interventions to treat drug refractory epilepsies by advancing our current understanding of the pathophysiology of epilepsy. New and exciting research tools such as chemogenetics provide innovative approaches in epilepsy research to meet these challenges. We begin with a history of chemogenetics followed by a description of the development of Designer Receptors Exclusively Activated by Designer Drugs (DREADDs) to provide a review on how these techniques are applied in epilepsy research.

### Chemogenetics Development

Chemogenetics can be defined as a method that confers cells with a pharmacological response to an engineered receptor (Lieb et al., 2019). The concept that a drug binds to specific sites or receptors on cell surfaces originates from Paul Ehrlich (1854 to 1915; Hill, 2006). Examples of modulating ion channels on the cell membrane of neurons date back to the same period (Cox and Gosling, 2014). In 1959, Nachmansohn isolated a protein identified as the first receptor activated by a neurotransmitter, the acetylcholine nicotinic receptor (Changeux, 2012; Cox and Gosling, 2014). Additional important pharmacological advancements included the development of methods to quantitatively measure affinity, efficacy, and the properties of agonists, partial agonists, and antagonists by the evaluation of functional responses in isolated tissues (Hill, 2006). These discoveries facilitated the development of the first chemogenetic tools. In 2001, Scearce-Levie et al. (2001) published an initial report of chemogenetics involving a receptor activated by an exogenous ligand. This was termed as receptors activated solely by synthetic ligands (Scearce-Levie et al., 2001). By genetically modifying an inhibitory κ-opioid receptor, they were able to generate two κ-opioid receptors, named Ro1 or Ro2, that no longer showed affinity to their endogenous ligand in heart tissue, dynorphin, but responded only to spiradoline, a selective κ-opioid agonist. The administration of spiradoline to mice expressing receptors activated solely by synthetic ligands in cardiac cells resulted in a decreased heart rate while having no effect in wild-type animals. The selectivity of spiradoline in activating only receptors activated solely by synthetic ligands in cardiac tissue may be explained by extremely low expression of endogenous κ-opioid receptors in the heart. In endogenous κ-opioid receptor-rich tissues such as the brain, the administration of spiradoline resulted in sedation of the animals and masked behavioral responses generated by the activation of Ro1 (Redfern et al., 1999; Scearce-Levie et al., 2001). The markedly sedative side effect of receptors activated solely by synthetic ligands precluded its application in neuroscience research and alternative chemogenetics methods were then developed.

To overcome the cross-reaction of the ligand with other endogenous receptors, Lechner et al. (2002) introduced a new approach in which cortical neurons were transfected with *Drosophila* allatostatin receptors. The allatostatin receptor is a non-mammalian G protein-coupled receptor (GPCR) that is solely activated by the insect peptide allatostatin. Delivery of allatostatin to *ex vivo* ferret brain slices induced hyperpolarization of mammalian cortical neurons expressing allatostatin receptors in a reversible manner without endogenous receptor cross-reaction (Lechner et al., 2002). However, allatostatin is a neuropeptide, so it likely does not cross the blood-brain barrier (BBB) when delivered systemically. This would require it to be delivered *via* intracerebroventricular injection for its application *in vivo*. Finally, Lerchner et al. (2007) demonstrated the viability of chemogenetics in freely moving animals by delivering a viral vector encoding for a modified heteromeric ivermectin gated chloride channel from *C. elegans*. Neuronal activity in the striatum of naïve mice was reversibly suppressed upon activation by ivermectin, a synthetic ligand that crosses the BBB (Lerchner et al., 2007).

### DREADDs Development

The predominant chemogenetic tools in epilepsy research are DREADDs (Lieb et al., 2019). Armbruster et al. (2007) introduced the concept of DREADDs by generating a family of GPCRs based on the human muscarinic acetylcholine receptor DREADD (hM_x_D_y_). GPCRs are the largest group of cell surface receptors in the central nervous system (Yang et al., 2021) and the Human Genome Project has identified more than 800 different GPCR genes (Hill, 2006). GPCRs are seven-transmembrane highly selective receptors that trigger intracellular signaling cascades through coupling to a range of intracellular proteins (G-proteins, β-arrestins, and kinases; Yang et al., 2021), and are grouped into four main families: G_i/o_, G_s_, G_q_, and G_12/13_ (Glukhova et al., 2018). GPCRs significantly contribute to regulation of neuronal excitability, and abnormalities in expression and activity of this class of receptors have been associated with different neuropathological processes including epilepsy (Yu et al., 2019). Therefore, GPCRs provided a logical platform for the development of DREADDs.

**Figure 1:**
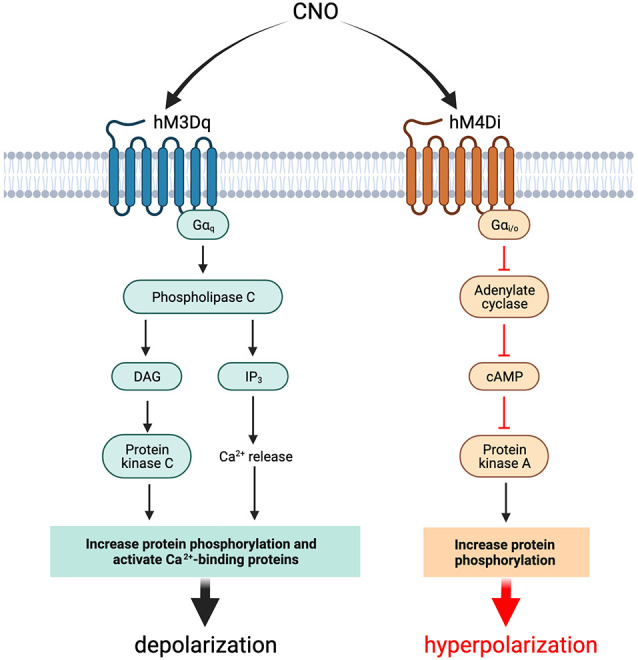
Effects of activation of hM3Dq and hM4Di with CNO in neurons and interneurons. Activation of the DREADD probe hM3Dq with CNO in neurons and interneurons results in cellular depolarization while activation of hM4Di results in hyperpolarization. The net effect of activation of the DREADD construct is dependent on whether inhibitory or excitatory chemogenetics are used and which cell populations are targeted. cAMP, cyclic adenosine monophosphate; CNO, clozapine-N-oxide; DAG, diacylglycerol; DREADDs, designer receptors exclusively activated by designer drugs; hM3Dq, G_q_-coupled human M3 muscarinic DREADD; hM4Di, G_i_-coupled human M4 muscarinic DREADD; IP_3_, inositol 1,4,5-trisphosphate.

The commonly used DREADDs in epilepsy research are hM3Dq and hM4Di. hM3Dq is an engineered muscarinic receptor coupled to a G_αq_ signaling cascade leading to neuronal excitation (Alexander et al., 2009) whereas hM4Di is coupled to G_αi_ and mediates neuronal inhibition (Armbruster et al., 2007; Stachniak et al., 2014). Both hM3Dq and hM4Di are irresponsive to their native ligand, acetylcholine, but have their intracellular signaling cascades mediated and activated solely by a pharmacologically inert and bioavailable compound, which is usually clozapine-N-oxide (CNO; Figure 1; Roth, 2016). Furthermore, DREADDs should have a minimal response in the absence of ligand binding. DREADDs activity typically relies upon a dose of CNO, which is usually in nanomolar concentrations.

However, CNO does not generate a cellular response in brain tissue when delivered systemically *in vivo*. Instead, CNO is reverse metabolized into clozapine that can cross the BBB and possesses high affinity to muscarinic DREADDs (Gomez et al., 2017). Despite the low concentrations of clozapine required to activate DREADDs, it is an atypical antipsychotic so CNO-derived clozapine may exert pharmacological effects in non-DREADDs targets that may result in undesirable behavioral changes. Therefore, the dose of the ligand must be titrated to control efficacy of treatment with DREADDs while mitigating potential side effects. When designing experiments, it is important to include appropriate control groups to assess for both: (*i*) potential side effects of CNO in non-DREADD-expressing animals; and (*ii*) the injection of a vehicle, such as saline, within DREADD-expressing subjects (MacLaren et al., 2016; Manvich et al., 2018). An alternative to relying on reverse metabolization of CNO is to inject clozapine at its much smaller reverse metabolized equivalent dose. Additionally, different ligands that possess higher affinity to DREADDs and reduced side effect profiles may be used, such as olanzapine, which is a second-generation atypical antipsychotic drug approved for use in humans by the Food and Drug Administration (Weston et al., 2019; Goossens et al., 2021).

### Methods Used to Drive DREADDs Expression

Selective expression of the engineered receptors in cell populations can be achieved by two main techniques: (*i*) intracerebral injection of adeno-associated virus (AAV) vectors encoding the engineered receptors to a genomic sequence, with the genomic sequence linked to cell-type specific transcription factors, which results in receptor expression in a targeted subset of cells; and (*ii*) using transgenic mice that express the engineered receptors in cell populations genetically defined by the Cre-driver mouse line (Alexander et al., 2009; Farrell and Roth, 2013). These two methods have been used to deliver DREADDs to targeted cell populations in a specific brain region. This allows the neuroscientists to investigate the role of these cells in seizure generation and propagation. The goal of this review is to summarize the existing literature where DREADDs are employed to improve our understanding of the pathophysiology of human epilepsy. The remainder of this review focuses on the application of DREADDs in epilepsy research.

## Materials and Methods

A PubMed-, Scopus-/ScienceDirect-, and Web of Science-based scoping review of chemogenetics in epilepsy research was performed. Combinations of queries used for searches were (“chemogenetic” OR “pharmaco-genetic” OR “DREADD” OR “hM4Di” OR “hM3Dq”) AND (“epilepsy” OR “seizure” OR “spasm” OR “epileptogenesis”). Publications that were classified as reviews were excluded. References were screened for additional relevant articles. Title/abstract and full text screenings were performed. Articles used for full text analysis were those using chemogenetics to investigate the pathophysiology of epilepsy.

## Results

**Figure 2:**
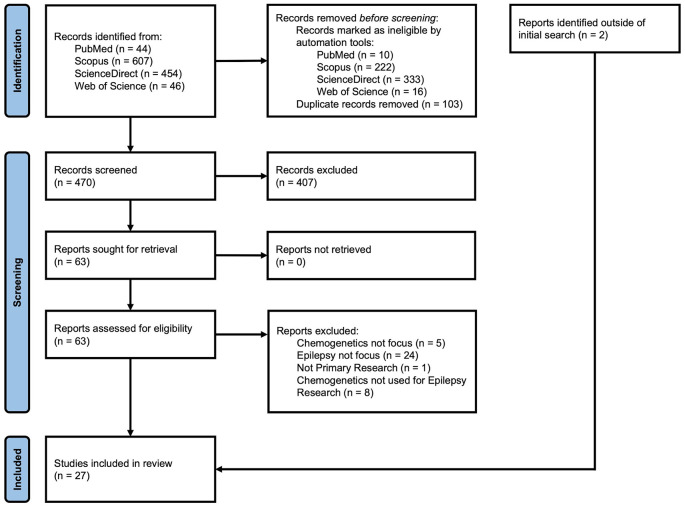
PRISMA flow diagram of record identification, screening, and inclusion process for this review.

Only primary or original research was considered in scope of this review. Our initial search returned 34 publications from PubMed, 385 from Scopus, 124 from ScienceDirect, and 30 from Web of Science. After removal of duplicates, 470 articles were included in our review. Publications were included if DREADDs were used to evaluate mechanisms of epileptogenesis or the epileptic phenotype. Additionally, publications were included if DREADDs were used to analyze biochemical and molecular mechanisms involved in epilepsy, even if results did not directly demonstrate seizure induction or seizure control. Publications were excluded if they evaluated seizures or spasms unrelated to epilepsy (e.g., alcohol withdrawal seizures) or focused purely on chemogenetics development (e.g., alternative ligands). After reading the abstract and applying inclusion and exclusion criteria, we identified 63 articles. After reading the full text and applying the same criteria, we included 25 articles in our analysis. Two (2) additional articles were identified by reference mining (i.e., citation chaining) resulting in a total of 27 articles. See Figure 2 for PRISMA flow diagram (Page et al., 2021). The information about the studies included in this review is summarized in Table 1 and reflects the structure of the sections on hippocampal and extrahippocampal networks. For each article in Table 1, we listed the DREADD construct. Four (4) studies that used DREADDs *in vitro* were excluded from the table but are included in the text. Three (3) publications on chemogenetics with potential for additional development were included in the “Discussion” Section.

**Table 1:** Responses of DREADDs targeted to hippocampal and extrahippocampal networks.

**Probe**	**Cellular specificity**	**Regional specificity**	**Cell type/Pathway**	**Animal model**	**Ligand**	**Figure Ref**.	**Reference**
**DREADDs Targeted to Hippocampal Networks**							
*Seizure Potentiation or Induction*							
hM3Dq	CaMKIIα promoter	AAV5 into DG bilaterally	DGC	C57BL/6 mice	CNO	1a (3)	Kahn et al. (2019)
	DrD2-Cre mice	AAV2 into HPC bilaterally	Mossy cells	Pilocarpine	CNO	1b (3)	Botterill et al. (2019)
	-	RV into DG bilaterally	Ectopic DGC	Pilocarpine C57BL/6 mice	CNO	1c (3)	Zhou et al. (2019)
hM4Di	AAV-Vgat-Cre	AAV2/8 into CA1	CA1 PV/SOM INs	HPC kindled ChAT-ChR2-YFP mice with CA1 fiber	CNO	1d (3)	Wang et al. (2020)
	PV-Cre mice	AAV2 into Sub	Sub PV-INs	Pentylenetetrazol	CNO	1e (3)	Drexel et al. (2017)
hM3Dq	CaMKIIα promoter	AAV into Sub	Sub PNs	Phenytoin-responsive amygdala kindled Wistar rats	CNO	1f (3)	Xu et al. (2019)
*Excitation to Decrease Seizures*							
hM3Dq	PV-Cre mice	AAV8 into HPC Bilaterally	HPC PV-INs	4-aminopyridine	CNO	2a (3)	Călin et al. (2018)
	PV-Cre mice	AAV into HPC	DG + CA3 PV-INs	Acute/chronic IHKA, and HPC kindled	CNO	2b (3)	Wang et al. (2018)
	SOM-Cre mice	AAV into CA1	CA1 PV/SOM-INs	Acute IHKA	CNO	2c (3)	Wang et al. (2020)
	Vgat-Cre mice	AAV into Sub	Sub PV/SOM-INs	Acute/chronic IHKA, and HPC kindled	CNO	2d (3)	Wang et al. (2017)
*Inhibition to Decrease Seizures*							
hM4Di	CaMKIIα promoter	AAV2/7 into HPC	HPC PNs	IPKA Sprague Dawley rats	Clozapine and Olanzapine	3a (3)	Goossens et al. (2021)
	CaMKIIα-Cre mice	AAV into HPC	DG-CA3 microcircuit	Acute/chronic IHKA, and HPC kindled	CNO	3b (3)	Wang et al. (2018)
	CaMKIIα-Cre mice	AAV into HPC	DG-CA3 microcircuit	HPC kindled	CNO	3b (3)	Chen L. et al. (2020)
	hSyn promoter	AAV8 into contralateral HPC	DG-CA3 microcircuit	CaMKIIa-ChR2 mice with DG diode	CNO	3b (3)	Berglind et al. (2018)
	CaMKIIα promoter	Recombinant AAV2/7 into ipsilateral HPC	DGC	IHKA C57BL/6 mice	CNO and Clozapine	3c (3)	Desloovere et al. (2019)
	POMC-Cre mice	-	DGC	Pilocarpine	CNO	3c (3)	Zhou et al. (2019)
	Nestin-CreER mice	-	Adult born DGC	TAM at 6 weeks and pilocarpine 2 weeks later	CNO	3c (3)	Zhou et al. (2019)
	-	RV into DG bilaterally	Ectopic DGC	Pilocarpine C57BL/6 mice	CNO	3d (3)	Zhou et al. (2019)
	-	RV into HPC	Ectopic DGC	Pilocarpine C57BL/6 mice	CNO	3d (3)	Lybrand et al. (2021)
	DrD2-Cre mice	AAV2 into HPC bilaterally	Mossy cells	Pilocarpine	CNO	3e (3)	Botterill et al. (2019)
	CaMKIIα promoter	AAV into Sub	Sub	Phenytoin-unresponsive amygdala kindled Wistar rats	CNO	3f (3)	Xu et al. (2019)
**DREADDs Targeted to Extrahippocampal Networks**							
*Seizure Potentiation or Induction*							
hM3Dq	CaMKIIα-tTA in TRE mice	-	HPC and cortex PNs	-	CNO	4a (4)	Alexander et al. (2009)
hM4Di	PV-Cre mice	-	HPC, somatosensory cortex, RTN, and cerebellar cortex PV-INs	-	-	4b (4)	Panthi and Leitch (2019)
*Excitation to Decrease Seizures*							
hM3Dq	PV-Cre mice	AAV into Motor cortex	Motor cortex PV-INs	Acute IHKA	CNO	5a (4)	Wang et al. (2018)
	ChAT-Cre mice	AAV2/8 into medial septum	Medial septum cholinergic neurons	Acute IHKA	CNO and Clozapine	5b (4)	Wang et al. (2020)
	Vgat-Cre mice	AAV into parafascicular nucleus of thalamus	Parafascicular nucleus INs	ChR2 right SNr with CA3 kindling	CNO	5c (4)	Chen B. et al. (2020)
*Inhibition to Decrease Seizures*							
hM4Di	CaMKIIα promoter	AAV into motor cortex	Motor cortex PNs	Pilocarpine and picrotoxin seizures, and tetanus toxin Epileptic rats	CNO	6a (4)	Kätzel et al. (2014)
	hSyn promoter	Recombinant AAV8 into midline thalamus bilaterally	Intralaminar thalamus neurons	Amygdala kindled Sprague Dawley rats	CNO	6b (4)	Wicker and Forcelli (2016)
	PV-Cre mice	AAV into SNr	SNr PV-INs	Acute/chronic IHKA	CNO	6c (4)	Chen B. et al. (2020)
	CRH-Cre mice	AAV into PVH bilaterally	PVH CRH neurons	Pilocarpine	CNO	6d (4)	Hooper et al. (2018)

**Figure 3:**
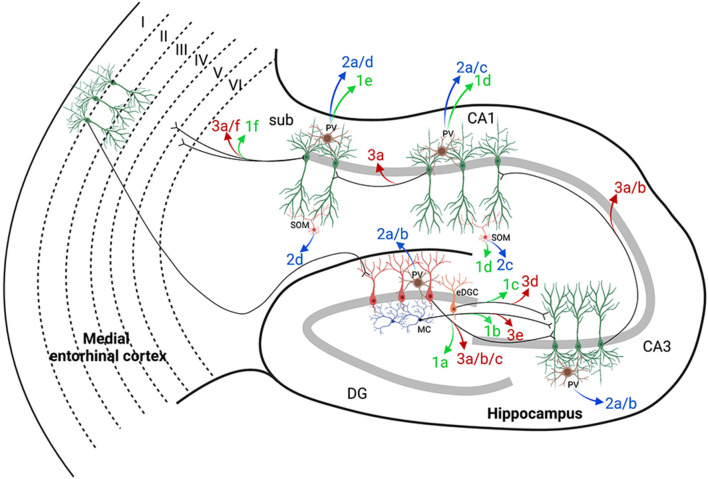
Hippocampal targets of chemogenetics interrogated by each of the authors cited. The two classically defined inputs from the entorhinal cortex (EC) to the hippocampus are the perforant pathway (PP; EC to DG) and the temporoammonic pathway (EC to CA1). A long excitatory synaptic loop (EC to DG to CA3 to CA1 to subiculum and EC) receives inputs from the PP by innervation of the dentate gyrus (DG) and is known as the trisynaptic pathway. Table 1 provides a key to the denoted projections and is organized by where each of the authors are cited in this review. Projections denoted in green were used by the authors cited to either potentiate or induce seizures with DREADDs (figure reference designation of 4). Blue labels indicate where hM3Dq was used to decrease seizures (figure reference designation of 5). Red labels indicate where hM4Di was used to decrease seizures (figure reference designation of 6). Projections labeled in black indicate connectivity that may contribute to the circuits evaluated. Created in BioRender.com. CA, Cornu Ammonis; DG, dentate gyrus; DREADDs, designer receptors exclusively activated by designer drugs; EC, entorhinal cortex; eDGC, ectopic dentate granule cells; hM3Dq, G_q_-coupled human M3 muscarinic DREADD; hM4Di, G_i_-coupled human M4 muscarinic DREADD; MC, mossy cells; PV, parvalbumin interneurons; SOM, somatostatin interneurons; Sub, subiculum.

*Table 1 reflects the structure of the sections on hippocampal and extrahippocampal networks. For each article, we listed DREADDs construct, respectively. For example, a figure reference of 1 refers to publications that used DREADDs to potentiate or induce seizures by targeting networks within the HPC and has a 3 in parenthesis because it is depicted in Figure 3. Publications that targeted similar cells or networks have the same alphabetical designation in the figure reference column. The cellular specificity column includes the viral promoter if it was used to drive cellular specificity rather than Cre driver mouse lines. AAV, adeno associated virus; CA, Cornu Ammonis; CaMKII, calcium/calmodulin-dependent protein kinase II; ChAT, choline acetyl-transferase; ChR2, channelrhodopsin-2; CNO, clozapine-N-oxide; CRH, corticotropin-releasing hormone; DG, dentate gyrus; DGC, dentate granule cells; DrD2, dopamine receptor D2; DREADDs, designer receptors exclusively activated by designer drugs; eDGC, ectopic dentate granule cell; ER, estrogen receptor; hM3Dq, G_q_-coupled human M3 muscarinic DREADD; hM4Di, G_i_-coupled human M4 muscarinic DREADD; HPC, hippocampus; hSyn, human synapsin 1; IHKA, intrahippocampal kainic acid; INs, interneurons; IPKA, intraperitoneal kainic acid; PN, pyramidal neuron; POMC, proopiomelanocortin; PV, parvalbumin; PVH, paraventricular hypothalamic nucleus; RTN, reticular thalamic nucleus; RV, retrovirus; SNr, Substantia nigra; SOM, somatostatin; Sub, subiculum; TA, temporoammonic; TAM, tamoxifen; TRE, tet response element; tTa, tet transactivator; Vgat; vesicular GABA transporter*.

We divided the articles included in this review into two main categories, i.e., those investigating hippocampal and extrahippocampal networks. Within the section on hippocampal networks, examples of seizure potentiation and induction by DREADDs are reviewed, followed by studies demonstrating seizure reduction using hM3Dq and then studies describing seizure reduction using hM4Di. A similar structure is used for the section on extrahippocampal targets, with examples of seizure induction described, followed by seizures reduced by hM3Dq and then by hM4Di. Finally, we discuss the experiments that use DREADDs to evaluate biochemical changes and then comorbidities associated with epilepsy.

### DREADDs Targeted to Hippocampal Networks

#### Seizure Induction by Targeting Hippocampal Networks

The most common form of seizure disorder in adults is temporal lobe epilepsy (TLE), which is characterized by a seizure onset zone located in the temporal lobe (Navidhamidi et al., 2017). The hippocampus contains excitatory networks that are important for many cognitive functions such as spatial learning and memory (Jokeit and Ebner, 2002; Sweatt, 2004; Strien et al., 2009; Buzsáki and Moser, 2013) and serve as a network substrate for the onset of TLE (Lothman et al., 1991; Chatzikonstantinou, 2014). Three primary excitatory loops, which connect hippocampal and parahippocampal structures, exist. A long trisynaptic loop from the entorhinal cortex (EC) to the dentate gyrus (DG) to area Cornu Ammonis (CA) 3 to area CA1 loops back to different layers of the EC *via* the subiculum. Two other loops that bypass the dentate gyrus are an intermediate-length loop from EC to CA3 to CA1 to subiculum/EC; and a short loop that projects directly from EC to CA1 (Ang et al., 2006; Coulter et al., 2011). In the normal condition, most of the afferent inputs from the cortex are filtered and tightly regulated by cells in the DG (granule cells) and/or area CA1 (pyramidal cells) which are thought to be the two most potent “inhibitory hubs” in the network (Ang et al., 2005; Coulter et al., 2011).

The publications reviewed in this section are summarized in Figure 3, which includes hippocampal networks with a key provided in Table 1 (figure reference designation of 1). The strategic location of the DG at the start of the trisynaptic pathway and the relative reluctance of dentate granule cells (DGCs) of the DG to fire led to postulation of the dentate “gate” hypothesis (Krook-Magnuson et al., 2015). Kahn et al. (2019) demonstrated the capability of DREADDs to induce seizures by providing evidence that non-epileptic mice expressing hM3Dq in calcium/calmodulin-dependent protein kinase II (CaMKIIα)-positive or excitatory neurons in the dorsal DG had variable behavioral seizures after administration of high doses of CNO (≥10 mg/kg). Although their report did not focus on seizure induction, their results provide a concept for further mechanistic studies.

An important means to investigate the role of a certain group of neuronal cells in generating seizures is to ask whether a change of their activities may potentiate seizures, i.e., increasing seizure probability when combined with a chemoconvulsant dose that does not cause seizures. Several groups have potentiated seizures resembling TLE by manipulating a subset of cells with hM3Dq or hM4Di. This has elucidated pathological mechanisms that contribute to the breakdown of the dentate gate. In addition to the classic DG to CA3 microcircuit, another target of DGCs is glutamatergic neurons located in the DG hilus adjacent to DGCs. These cells are known as mossy cells and have been demonstrated to innervate both DGCs and parvalbumin-expressing basket cells, which are inhibitory γ-aminobutyric acid-releasing (GABAergic) interneurons (INs) in the DG (Scharfman and Myers, 2013). To explore the excitatory role of mossy cells in epileptogenesis, Botterill et al. (2019) selectively expressed hM3Dq in dopamine receptor D2-Cre^+/–^ mice. These animals preferentially expressed Cre recombinase in mossy cells. They showed that activation of hM3Dq in mossy cells decreased latency to convulsive seizures after pilocarpine injection (Botterill et al., 2019). Contrary to the prior hypothesis that mossy cells prevent epilepsy, these results suggested that mossy cells become seizure inducing during pathological conditions by increasing excitation of DGCs while not changing their innervation of basket cells. Alternatively, basket cells like other GABAergic INs may become dysfunctional upon sustained excitation.

Another group of cells in the DG that have been investigated for their role in seizure generation are newborn DGCs, which are developed from neural stem cells. In the granular cell layer of the DG, neurogenesis persists throughout life in the adult hippocampus. In physiological conditions, newborn DGCs integrate into existing hippocampal circuitry, which is necessary for hippocampus-dependent learning and memory processes (Parent, 2007). These newborn DGCs may contribute to the breakdown of the dentate gate in TLE. To investigate this, Zhou et al. (2019) expressed hM3Dq in DGCs born 3 days after pilocarpine-induced status epilepticus (SE). They demonstrated that CNO-mediated activation of newborn DGCs two and a half months after transfection with hM3Dq resulted in epileptic spikes and spontaneous recurrent seizures, which are characteristics of chronic epilepsy. Furthermore, they demonstrated that newborn DGCs ectopically integrated into the trisynaptic pathway forming recurrent excitatory loops and contributed to increases in DG excitability (Zhou et al., 2019).

Another means to potentiate TLEs is to directly target the main source of inhibitory signaling in the brain, GABAergic INs. GABAergic INs discussed in this review may be further categorized as parvalbumin (PV)-, somatostatin (SOM)-, cholecystokinin-, and vasointestinal peptide-expressing INs (Pelkey et al., 2017; Marafiga et al., 2020). Under physiological conditions, GABAergic INs function to restrain excessive excitation in principal neurons *via* feedforward, feedback, or tonic inhibitory mechanisms (Marafiga et al., 2020). The breakdown of this inhibition results in a disturbance of the excitation/inhibition balance that contributes to the generation of seizures (Magloire et al., 2019). Wang et al. (2020) used hM4Di to block inhibition mediated by optogenetic stimulation of cholinergic neurons in the medial septum in intrahippocampal kainic acid (IHKA)-treated mice. The hM4Di-mediated inhibition of PV- or SOM-INs in CA1 resulted in increased seizures, suggesting that these INs were the downstream effectors of medial septum cholinergic neurons (Wang et al., 2020). These results suggest a possible involvement of dysfunctional PV- and SOM-INs in contributing to the generation of seizures and demonstrate the ability to target specific subsets of INs with a DREADD construct.

While the dentate gate restrains excitatory input, the other “gate” of the hippocampus is the subiculum which receives information from hippocampal area CA1 by both the trisynaptic pathway and temporoammonic pathway (Coulter et al., 2011). To evaluate the effect of the loss of inhibition at this node, Drexel et al. (2017) evaluated the transient inhibition of PV-INs in the subiculum using hM4Di. While transient inhibition of PV-INs did not generate seizures in non-epileptic mice, the injection of CNO combined with a sub-convulsant dose of pentylenetetrazol induced mice to show clusters of spike-wave discharges (Drexel et al., 2017). Similarly, Xu et al. (2019) used a DREADD construct to increase phenytoin resistance in epileptic mice by activating hM3Dq expressed in subicular pyramidal neurons (PNs). Contrary to phenytoin increasing afterdischarge threshold in wild-type mice, CNO in combination with phenytoin resulted in decreased afterdischarge threshold. These studies support a critical role of the subiculum in gating excitatory transmission.

#### Seizure Control by hM3Dq Targeted to Hippocampal Networks

In contrast to the previous subsection where we described publications that used DREADDs to induce or potentiate seizures, this section presents *in vitro* and *in vivo* experiments where researchers targeted hM3Dq to hippocampal networks to suppress epileptic seizures by increasing inhibitory signaling. The publications reviewed in this section are summarized in Figure 3, which includes hippocampal networks with a key provided in Table 1 (figure reference designation of 2). A common strategy to suppress seizures has been the potentiation of GABAergic signaling (Perucca and Mula, 2013). The GABA_A_ receptor is a common target of ASDs and it has been demonstrated that the antiseizure mechanism of benzodiazepines and barbiturates is by direct action on GABA_A_ receptors (Greenfield, 2013). Therefore, selective targeting of INs to constrain hyperexcited networks is a logical choice for the application of hM3Dq. Several groups have demonstrated the feasibility of harnessing GABAergic INs to suppress seizure activity in models of TLE (Wang et al., 2017, 2018, 2020; Călin et al. 2018).

Since there are different subtypes of GABAergic INs, it is likely important to determine which class of INs promote the most efficacious inhibitory effect on excitatory neurons in the hippocampus. To evaluate this, Călin et al. (2018) selectively targeted PV-, SOM-, and vasointestinal peptide-INs with hM3Dq *in vitro* using organotypic hippocampal slice cultures. Their data suggested that when targeting the entire hippocampus, PV-INs are more efficacious in suppressing epileptiform activity than other types of INs. Furthermore, the selective activation of hippocampal PV-INs with hM3Dq reduced the severity of systemic 4-aminopyridine-induced seizures in mice (Călin et al. 2018). This report demonstrated the relative difference in the effect of hM3Dq when manipulating subpopulations of INs, which is an important tool in investigating the role of specific cells in controlling seizures.

Since hyperexcitability of DGCs is associated with the emergence of seizures in TLE (Kahn et al., 2019), activating PV-INs within DG and CA3 subfields may also be efficacious in controlling seizures. In addition to confirming this hypothesis, Wang et al. (2018) showed that the anti-seizure effect of hM3Dq occurred in a CNO dose-dependent manner in PV-Cre mice treated with IHKA. An increased dose of CNO activated hM3Dq-expressing PV-INs in the DG and CA3 resulting in increased latency to SE, and decreased duration of chronic seizures and animal mortality. Furthermore, they demonstrated that activation of ventral hippocampal PV-INs reduced the number and duration of generalized seizures in mice during an 8-h measurement window following each dose of CNO for three consecutive days. The authors then applied the same technique to electrically kindled animals and showed that the CNO delayed seizure progression and decreased the duration of generalized seizures among these animals (Wang et al., 2018). In a later study, Wang et al. (2020) provided evidence in their supplemental data comparing direct hM3Dq-mediated activation of PV- and SOM-INs located in CA1. In CA1, these INs receive excitatory inputs from EC through the temporoammonic pathway and from CA3 through Schaffer collaterals, so area CA1 may also be an ideal node for targeting with hM3Dq to control seizures. In addition to decreasing seizures in an acute IHKA model, the study revealed that SOM-INs in CA1 have a greater inhibitory effect on this circuit than PV-INs (Wang et al., 2020).

In the subiculum, Wang et al. (2017) demonstrated that selective hM3Dq-mediated activation of GABAergic INs of vesicular GABA transporter-Cre mice resulted in delayed generalization of IHKA induced SE and reduced episodes of generalized seizures. Vesicular GABA transporter-Cre mice express Cre recombinase under control of the vesicular GABA transporter promoter in both PV- and SOM-INs. Interestingly, the excitation of these INs during the chronic phase of IHKA-induced epilepsy resulted in a transient twofold increase in the duration of generalized seizures during the 3-day CNO treatment window as compared to 3 days prior and post treatment (Wang et al., 2017). Their results suggest the involvement of a phenomenon known as ionic plasticity that may occur during epileptogenesis. This phenomenon refers to a shift from an inhibitory to an excitatory signaling profile of GABA in principal neurons by the modulation of neuronal functions through changes in GABAergic driving forces caused by long-term impairments of ion-regulatory molecules such as cation-chloride cotransporters (K-Cl cotransporter—KCC2, Na-K-2Cl cotransporter—NKCC1), Na-K ATPase, and carbonic anhydrase (Kaila et al., 2014). This early finding suggests that inhibitory neurons in the subiculum may display different roles in seizure generation and modulation in the different phases of epileptogenesis.

#### Seizure Control by hM4Di Targeted to Hippocampal Networks

Since the potentiation of GABA signaling with hM3Dq was demonstrated to be effective in constraining seizures in the previous section, in this section we address whether directly targeting hM4Di to PNs within a hippocampal seizure onset zone is as efficient in preventing and stopping seizures. This method was pioneered in epilepsy research by Armbruster et al. (2007) when they expressed hM4Di in hippocampal neurons in culture and demonstrated that administration of CNO induced selective membrane hyperpolarization and neuronal inhibition. Later, Avaliani et al. (2016) used a valproate-refractory model of epilepsy to provide evidence that inhibition of PNs of brain slices in organotypic culture could reduce electrically evoked seizure activity. They demonstrated that hyperpolarization of CA3 excitatory neurons by hM4Di was sufficient to suppress DG-initiated stimulus train-induced bursting (Avaliani et al., 2016). These *in vitro* studies represent proof-of-concept models that may be used prior to moving hM4Di to *in vivo* models. The studies presented here on in this section are summarized in Figure 3, which includes hippocampal networks with a key provided in Table 1 (figure reference designation of 3).

Goossens et al. (2021) expanded upon the *in vitro* experiment introduced by Armbruster et al. (2007) by targeting all hippocampal excitatory neurons *in vivo*. They demonstrated that hM4Di-mediated inhibition of these neurons by a single or repetitive (6-h interval) subcutaneous clozapine injection resulted in decreased acute seizure frequency in rats with epilepsy induced by systemic KA. Additionally, clozapine or olanzapine were infused continuously for 7 days using osmotic minipumps, which resulted in significant seizure suppression during the first 4–5 days of treatment. However, seizure frequency increased to pre-treatment levels in the last 2 days of treatment with seizure duration exceeding baseline 3–4 days into treatment. Furthermore, after the removal of the minipumps, the animals showed a rebound effect both in seizure frequency and duration, reaching levels above the baseline observed before the onset of the treatment with CNO (Goossens et al., 2021). This phenomenon is comparable to tolerance effects observed with ASD usage in treating patients with seizure disorders. Tolerance effects of DREADDs should be considered when applying them to epilepsy research in general. Tapering the dosage of ligand instead of abrupt discontinuation could potentially reduce the rebound effect observed by Goossens et al. (2021). Alternatively, targeting DREADDs to more specific subsets of neurons in the hippocampus may remove the observed desensitization, which is similar to the “honeymoon effect” (i.e., loss of efficacy of ASD) observed in some patients after continued treatment with an ASD (Löscher and Schmidt, 2006). Regardless, the results of Goossens et al. (2021) highlight the importance of non-DREADD expressing controls treated with ligand for interpretation of results.

More anatomically specific inhibition was demonstrated by Wang et al. (2018) when they directly inhibited hippocampal PNs expressing hM4Di in the DG and CA3 ipsilateral to IHKA injection in urethane-anesthetized mice. This led to decreased neuronal firing. Subsequently, they showed that the administration of CNO once per day for 3 days in freely moving mice induced the inhibition of PNs in the DG and area CA3, which in turn resulted in increased latency to seizure progression, decreased seizure duration, and decreased number of generalized seizures during the 8 h of recording after CNO treatment. Although no statistic was provided, a trend toward rebound hyperexcitation after CNO treatment was discontinued was presented in their data. Furthermore, the receptor desensitization described by Goossens et al. (2021) was not observed, which may be due to differences in regional specificity of hM4Di expression, ligand dosing schemes, or lengths of experiments. Additional experiments are necessary to determine the source of the variability in results (Wang et al., 2018). Similar results on seizure progression were reproduced by Chen L. et al. (2020) using the electrical kindling model.

CA3 neurons may project directly (Witter, 2007) or secondarily from mossy cells (Scharfman and Myers, 2013) to the contralateral CA3 and CA1, and it has previously been demonstrated that bilateral DG activation is required for progression of afterdischarge durations (Stringer and Lothman, 1992). hM4Di has also been employed to investigate the role of excitatory neurons and their implications in pathological changes in transhemispheric neuronal networks. Berglind et al. (2018) employed optogenetics to generate focal afterdischarges and expressed hM4Di among PNs in the contralateral DG and CA3. The activation of these PNs by CNO decreased the duration of afterdischarges (Berglind et al., 2018). However, Krook-Magnuson et al. (2015) previously demonstrated that optogenetic inhibition of contralateral DGCs was insufficient to inhibit seizures, so additional experiments are necessary to determine if persistent manipulation of contralateral DG and CA3 neurons with hM4Di would reduce seizures.

Since optogenetic inhibition of DGCs ipsilateral to IHKA was previously demonstrated to inhibit seizures (Krook-Magnuson et al., 2015), prolonged suppression of DGCs expressing hM4Di may result in a similar inhibitory effect. Desloovere et al. (2019) evaluated inhibition of CaMKIIα PNs in the DG. They suggested that DGCs ipsilateral to IHKA injection were predominantly transfected, demonstrated modulatory results of hM4Di on inhibiting seizures, and evaluated long-term effects of inhibition. Specifically, they used either 3 or 10 mg/kg of CNO, or the CNO equivalent doses of clozapine (0.03 or 0.1 mg/kg respectively) to inhibit these DGCs. Their results showed that similar relative ligand concentrations resulted in similar levels of inhibition on seizures. Furthermore, their results revealed that the seizure suppressive effect of these doses of clozapine had a duration of action greater than 8 h. In a subsequent experiment, they demonstrated that chronic repeated administration of the same doses of clozapine once every 8 h for 3 days was capable of near complete suppression of seizure activity during the previously established duration of effect. However, a trend toward rebound hyperexcitability was observed a day after the last dose (Desloovere et al., 2019).

As described previously, newborn DGCs are derived from neural stem cells and integrate ectopically into the trisynaptic pathway forming excitatory loops, contributing to breakdown of the dentate gate. Therefore, hM4Di may be used to control seizures by inhibiting cells derived from neural stem cells. First, Zhou et al. (2019) replicated the anti-seizure effect of inhibiting all DGCs. They then demonstrated that long-term inhibition of neural stem cells that integrate into hippocampal networks could reduce pilocarpine-induced recurrent seizures. Next, they used double-transgenic mice that expressed Cre recombinase fused to an estrogen receptor only in Nestin-positive neural stem cells. Tamoxifen was administered to the mice 2 weeks prior to pilocarpine injection resulting in the expression of hM4Di in the cells derived from these stem cells. Upon development of spontaneous seizures two and half months later, the inhibition of these hM4Di-expressing cells with CNO injected every 8 h for 3 days resulted in decreased frequency of epileptic spikes and spontaneous recurrent seizures. This effect on seizures receded after the completion of treatment with CNO. They suggested that only neural stem cells that have differentiated into DGCs contribute to anti-seizure effects; however, neural stem cells integrate into regions other than the DG hilus so they performed an additional experiment to specifically inhibit newborn DGCs. They injected a retrovirus coding for hM4Di 3 days after pilocarpine injection. Upon development of spontaneous seizures, they observed a reduction in seizure frequency on the day of CNO injection (Zhou et al., 2019). Lybrand et al. (2021) further investigated this mechanism by examining the anatomic changes associated with manipulations of newborn DGCs and their correlations to the occurrence of spontaneous seizures. To do this, animals expressing hM4Di in newborn DGCs were injected intraperitoneally with pilocarpine, followed by CNO administered once daily for the 2 weeks following SE. Eight weeks after the last CNO injection, they observed a significant reduction in ectopic newborn DGCs and reduced seizure frequency. Furthermore, area CA3 back-projections, a part of newborn DGC networks, were reduced while EC projections were increased from non-hM4Di controls toward non-pilocarpine controls (Lybrand et al., 2021). Botterill et al. (2019) harnessed another pathophysiological mechanism when they selectively inhibited hM4Di-expressing mossy cells in the DG of dopamine receptor D2-Cre^+/–^ mice with CNO prior to inducing SE with pilocarpine. Their results demonstrated that, in addition to attenuating SE and decreasing neurodegeneration in the hilus and CA3, the inhibition of mossy cells in the DG resulted in reduced number, frequency, and severity of recurrent seizures during the chronic seizure phase (Botterill et al., 2019).

Previously in this review, we discussed results obtained by Xu et al. (2019) that showed that epileptic mice had increased resistance to phenytoin after activation of hM3Dq-expressing PNs in the subiculum. However, in the same publication, Xu et al. (2019) also decreased phenytoin resistance in the same phenytoin-resistant mouse model by expressing hM4Di in subicular PNs. Specifically, their results demonstrated that the administration of CNO along with phenytoin raised the afterdischarge threshold in mice that were kindled by the electrical stimulation of the amygdala. Interestingly, the inhibitory effect of hM4Di did not increase the afterdischarge threshold in the absence of phenytoin (Xu et al., 2019). Since phenytoin was administered intraperitoneally, it likely affected cells outside of the hippocampus. The requisite of systemic phenytoin for efficacy of specific inhibition of the subiculum suggests a more complex circuit interaction involving global networks when the seizure focus is located outside of the hippocampus. Targeting a single node within an excited network may not be sufficient to abrogate seizures. Furthermore, choke points distal to the focus may be more efficient in gating excessive network activity (Paz and Huguenard, 2015). For this reason, other researchers have focused their efforts on evaluating the impact of extrahippocampal networks and nodes on epileptic activity. In the next section, we present publications that used DREADDs to investigate extrahippocampal networks.

### DREADDs Targeted to Extrahippocampal Networks

#### Seizures Induced by Targeting Extrahippocampal Networks

**Figure 4:**
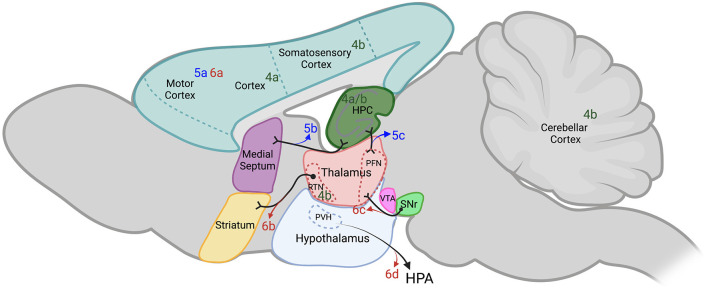
Extrahippocampal targets of chemogenetics interrogated by each of the authors cited. Sagittal section of adult rodent brain. Table 1 provides a key to the denoted publications and is organized by where each of the authors are cited in this review. Publications denoted in green were used by the authors cited to either potentiate or induce seizures with DREADDs (figure reference designation of 4). Blue labels indicate where hM3Dq was used to decrease seizures (figure reference designation of 5). Red labels indicate where hM4Di was used to decrease seizures (figure reference designation of 6). Projections labeled in black indicate connectivity that may contribute to the circuits evaluated. Dotted lines indicate structures outside of the plane depicted. Created in BioRender.com. DREADDs, designer receptors exclusively activated by designer drugs; hM3Dq, G_q_-coupled human M3 muscarinic DREADD; hM4Di, G_i_-coupled human M4 muscarinic DREADD; HPA, hypothalamic-pituitary-adrenal axis; HPC, hippocampus; PFN, parafascicular nucleus; PVH, paraventricular hypothalamic nucleus; RTN, reticular thalamic nucleus; SNr, substantia nigra pars reticulata; VTA, ventral tegmental area.

To begin, we will describe studies where extrahippocampal networks were manipulated using DREADDs to create new seizure models. Despite the prevalence of using chemoconvulsants in seizure models, they have several drawbacks including lack of control over the interval between the administration of chemoconvulsants and seizure onset, variability in the drug metabolism, and possible off-target and unintended side effects (Cela and Sjöström, 2019). The use of DREADDs to develop seizure animal models may mitigate these drawbacks. Figure 4 and Table 1 (figure reference designation of 4) summarize extrahippocampal circuits discussed in this section.

The first example of the use of DREADDs to induce seizures with probes targeted to extrahippocampal networks was demonstrated by Alexander et al. (2009) when they developed a mutant mouse line that expressed hM3Dq in CaMKIIα-positive neurons in the hippocampus and throughout the cortex. The systemic administration of at least 1 mg/kg of CNO in this mutant mouse line induced neuronal activation and generalized seizures similarly observed in chemoconvulsant-induced animal models of acute epilepsy. Additionally, the authors showed that there is a dose dependent response to CNO in seizure severity and the percentage of animals reaching SE (Alexander et al., 2009).

One third of all epilepsies have a genetic origin known as idiopathic generalized epilepsies. Treatment of idiopathic generalized epilepsies with ASDs may be ineffective. Additionally, patients with idiopathic generalized epilepsies often present with a structurally normal brain on imaging studies and have no focal seizure onset zone. This in turn renders the patient ineligible for resective surgery [Engel and International League Against Epilepsy (ILAE), 2001; Mullen et al., 2018]. Absence epilepsy is a non-convulsive idiopathic generalized epilepsy believed to arise from cortico-thalamocortical circuitry and is characterized by spike-wave discharges (McCormick and Contreras, 2001). Panthi and Leitch evaluated the effect of global and focal inhibition of hM4Di-expressing extrahippocampal PV-INs in double transgenic non-epileptic mice. They achieved network-wide inhibition of all cortico-thalamocortical PV-INs by intraperitoneal injection of CNO doses of 5.0 mg/kg or greater. The inhibition induced paroxysmal oscillatory activity known as afterdischarges. Inhibition of somatosensory cortex or reticular thalamic nucleus PV-INs was also achieved by focal injection to the somatosensory cortex or reticular thalamic nucleus, respectively. This approach required lower CNO doses of approximately 2.5 mg/kg and resulted in afterdischarges, spike wave discharges, and behavioral changes characteristic of absence seizures. The study suggested that the somatosensory cortex and reticular thalamic nucleus PV-INs restrained cortical pyramidal cells and thalamic cortical cells, respectively (Panthi and Leitch, 2019).

Although the CNO doses required to generate seizures are much higher than the normal effective doses (≅1 mg/kg) for DREADDs activation, these results show the potential of generating acute generalized seizures in an “on demand” way with high cellular selectivity. By targeting hM3Dq or hM4Di to specific regions and subsets of cells, DREADDs reduce off-target effects of chemoconvulsants since CNO is inert to receptors other than the expressed DREADD. Furthermore, the effect of DREADDs may be titrated both at the level of receptor expression and dose of ligand administered, which may allow for tighter control over the severity and duration of seizures produced in animals.

#### Seizure Control by hM3Dq Targeted to Extrahippocampal Networks

In addition to activating cells within the hippocampus, hM3Dq-mediated excitation has allowed for evaluation of extrahippocampal neuronal circuits on seizures originating from within the hippocampus. The studies presented in this section are summarized in Figure 4 and Table 1 (figure reference designation of 5). Wang et al. (2018) evaluated hM3Dq-mediated activation of motor cortex PV-INs in the same IHKA epilepsy model presented previously. Their data shows that direct activation of motor cortex PV-INs increased the latency to SE but did not reduce the number of generalized seizures. This may suggest that hippocampal inhibitory cells are a better target for reducing TLE progression than cortical areas (Wang et al., 2018). In another study, the same investigators shifted their attention to activation of another extrahippocampal network, the medial septum to hippocampus cholinergic circuit. They demonstrated that administration of CNO to choline acetyl-transferase-Cre mice expressing hM3Dq in medial septum cholinergic neurons resulted in reduced number and duration of spontaneous seizures in epileptic mice subjected to the same IHKA model of TLE. Since the muscarinic agonist pilocarpine is a commonly used chemoconvulsant, exciting cholinergic neurons to inhibit seizures is potentially counterintuitive. Therefore, Wang et al. (2020) used retroviral tracing to demonstrate that most of these cholinergic neurons projected directly to PV- and SOM-INs in the hippocampus. We have previously described that activation of hippocampal PV- and SOM-INs with hM3Dq has an inhibitory effect on seizures. However, they found that in these choline acetyl-transferase-Cre mice expressing hM3Dq in the medial septum, the sustained antiseizure effect generated by the administration of a daily single dose of CNO for 7 days was maintained for the 7 days following the end of CNO treatment. This may suggest that synaptic plasticity was induced from the sustained modulation of cholinergic signaling (Wang et al., 2020).

The parafascicular nucleus of the thalamus is known to be involved in the generation of physiological oscillatory rhythms and in the control of epileptic seizures due to its projections from and to areas implicated in seizure generation such as the cortex, thalamus, hippocampus, and especially the striatum (Vuong and Devergnas, 2018). In an elegant study using the IHKA animal model of spontaneous seizures in vesicular GABA transporter-Cre mice, Chen B. et al. (2020) demonstrated that the activation of parafascicular nucleus GABAergic neurons with hM3Dq prior to the injection of KA nearly doubled the latency to seizure generalization and decreased the number of generalized seizures. Mortality was also reduced in these mice. Furthermore, retrograde tracing provided evidence that GABAergic neurons in the substantia nigra pars reticulata were upstream of the parafascicular nucleus GABAergic neurons. Optogenetic activation of GABAergic neurons expressing channelrhodopsin-2 in the substantia nigra of vesicular GABA transporter-Cre mice potentiated kindling effects from electrical stimulation of CA3. Activation of hM3Dq-expressing GABAergic neurons in the parafascicular nucleus with CNO prior to kindling events removed this optogenetic pro-kindling effect. This suggests that GABAergic neurons in the substantia nigra modulate the antiseizure effect of parafascicular nucleus GABAergic neurons (Chen B. et al., 2020).

#### Seizure Control by hM4Di Targeted to Extrahippocampal Networks

For the motor component of a seizure to manifest, it most likely must generalize to regions of the brain that control movement. Additionally, extrahippocampal systems may alter hippocampal excitability through direct or indirect connections. We now present publications that constrained extrahippocampal cells or networks with hM4Di to control seizures (summarized in Figure 4 and Table 1—reference designation of 6). Evidence suggests that seizure control can be achieved by the direct inhibition of motor neurons. Kätzel et al. (2014) demonstrated that direct inhibition of PNs of the motor cortex by hM4Di reduced acute motor seizures induced by pilocarpine and picrotoxin. In their experiments, picrotoxin-induced behavioral seizures were reduced only after 3 months which allowed for maximal hM4Di expression. Additionally, they demonstrated the capability of inhibition of motor neurons in reducing seizure severity over an extended course of more than 3 h in the tetanus-toxin model of neocortical epilepsy (Kätzel et al., 2014).

Since the epileptic focus is not always defined, hM4Di was also employed to evaluate if the thalamus could act as a chokepoint to networks involved in epilepsy. In a study performed by Wicker and Forcelli, the inhibition of thalamic neurons by hM4Di resulted in limbic seizure attenuation in rats submitted to electrical kindling in the amygdala. Furthermore, they showed that the level of reduction in seizure severity and duration of electrographic activity was dependent on the CNO dose administered. Additionally, higher doses of CNO (10 mg/kg) completely blocked seizure activity in a subset of animals (Wicker and Forcelli, 2016). In another previously discussed thalamic interface, the direct inhibition of PV-INs expressing hM4Di in the substantia nigra pars reticulata of PV-Cre mice resulted in prolonged latency to KA-induced SE and seizure generalization, while chronic treatment was able to reduce the number and severity of spontaneous seizures (Chen B. et al., 2020).

The hypothalamic-pituitary-adrenal axis is under control of the paraventricular nucleus of the hypothalamus. Corticotropin-releasing hormone neurons in the paraventricular nucleus of the hypothalamus may provide a link between increased corticosteroid levels and seizure susceptibility (Herman et al., 2003). Previous publications have demonstrated that increased corticotropin-releasing hormone increased PN excitability in the hippocampus (Aldenhoff et al., 1983; Hollrigel et al., 1998; Maguire and Salpekar, 2013). To further investigate the connection between stress and seizures, Hooper et al. (2018) bilaterally inhibited corticotropin-releasing hormone neurons in the paraventricular nucleus of the hypothalamus by expressing hM4Di in transgenic corticotropin-releasing hormone-Cre mice. Orally administered CNO led to a reduction in pilocarpine-induced seizures while also reducing behaviors associated with depression (Hooper et al., 2018). Corticotropin-releasing hormone neurons are under tight control of GABA_A_ INs from multiple limbic brain regions including the hippocampus and thalamus (Cullinan et al., 1993). However, balanced levels of corticosterone are required for physiological function of the hippocampus (Diamond et al., 1992). Therefore, an abnormal hippocampus may lead to hyperactivity of the hypothalamic-pituitary-adrenal axis and amplify the pro-ictal effect of corticotropin-releasing hormone, further propagating the breakdown of homeostatic mechanisms.

### The Use of DREADDs to Understand Biochemical Mechanisms of Epilepsy

In addition to anatomical/network changes, one critical characteristic of epilepsy that results from a persistent hyperexcitable network is the induction of maladaptive responses at the cellular level (Queenan et al., 2018). One of the most important cellular alterations is known as Hebbian plasticity, which comprises mechanisms of long-term potentiation and long-term depression. These mechanisms drive long-lasting alterations in synaptic strength to ensure network stability, providing feedback to unrestrained network hyperactivation (Lignani et al., 2020). Pathological mossy fiber sprouting may occur after death of DGC terminals (Cavarsan et al., 2018) and is assumed to contribute to recurrent closed-loop circuitry of excitatory synapses between the DG and CA3 (Santhakumar et al., 2005; Buckmaster, 2010). To better understand the synaptic homeostatic mechanisms between the hippocampal layers CA3 and DG, Queenan et al. (2018) investigated the synaptic homeostatic plasticity resulting from epileptogenesis in mice. In their report, they focused on hM4Di-expressing DGCs with mossy fibers that targeted CA3 neurons that were not transfected with hM4Di. Chronic inactivation of this subset of DGCs with CNO was associated with cellular changes in these untransfected CA3 neurons including large increases in presynaptic bouton size, containing increased synaptoporin, and tripled the postsynaptic accumulation of the major scaffolding protein of mature glutamate synapses, postsynaptic density protein-95. Their results suggest that presynaptic mechanisms drive both pre-and postsynaptic expansion of DG-CA3 synapses (Queenan et al., 2018). In addition to a key role in epileptogenesis, these synaptic mechanisms associated with the accumulation of postsynaptic density protein-95 have been described to be fundamental to cognitive and behavioral functions in both healthy and pathological conditions (Yao et al., 2004; Delint-Ramírez et al., 2008; Keith and El-Husseini, 2008; Sun et al., 2009; Coley and Gao, 2019).

As discussed previously, DGCs have low excitability, which contributes to the gating function of the DG. In excitatory neurons, the KCC2 channel is necessary for proper function of postsynaptic GABA_A_ receptor signaling and hyperpolarizing GABAergic transmission. KCC2 functions by maintaining low intracellular Cl^−^ concentration in normal conditions through inward K^+^ gradients for the extrusion of Cl^−^ (Cristo et al., 2018). The downregulation of KCC2 can lead to increased neuronal excitability associated with numerous psychiatric and neurologic disorders including epilepsy (Kahle et al., 2016; Chen et al., 2017; Duy et al., 2019; Goutierre et al., 2019). Therefore, the downregulation of KCC2 may lead to increased excitability in DGCs and the breakdown of the dentate gate. First, to test whether the downregulation of KCC2 can be reversed or attenuated, Goutierre et al. (2019) used KCC2-directed small hairpin RNA to downregulate KCC2 expression in the hippocampus of rats expressing hM4Di in DGCs. Their data suggests that the knockdown of KCC2 in DGCs (with similar results in CA1) resulted in reduced potassium conductance due to diminished expression of outward rectifying Task-3 potassium channels, leading to strengthened EC afferents and hippocampal hyperexcitability. After treatment of these rats with CNO, the researchers observed a restoration in DGC membrane properties, which reversed the hyperexcitability generated by KCC2 knockdown. However, selective KCC2 knockdown in the DG of rats did not result in spontaneous recurrent seizures and did not potentiate the effects of pilocarpine induced SE (Goutierre et al., 2019).

The dysregulation of glial cells, specifically astrocytes, has also been implicated in the generation and worsening of epileptiform activity by the release of excitatory gliotransmitters including glutamate, D-serine, and ATP (Robel et al., 2015; Vargas-Sánchez et al., 2018). Therefore, an application of hM3Dq was to modulate astrocytes to investigate both the intrinsic mechanisms of reactive astrogliosis and their influence on surrounding neurons. Durkee et al. (2019) expressed hM3Dq in astrocytes of the hippocampus by using a glial fibrillary acidic protein promoter. The selective activation of these astrocytes increased extracellular Ca^2+^ and facilitated glutamate release, which activated N-methyl-D-aspartate receptors and induced slow inward currents in surrounding neurons. Interestingly, selective activation of both hM3Dq and hM4Di in astrocytes in the primary somatosensory cortex of mouse brain slices generated increased glutamate release resulting in enhanced neuronal excitability (Durkee et al., 2019).

### Using DREADDs to Investigate the Comorbidities of Epilepsy

Since the progression of epilepsy exposes the brain to prolonged abnormal electrical activity, it may lead to cognitive and psychosocial impairments (Fisher et al., 2014; Falco-Walter et al., 2018). The most prominent cognitive problems found in TLE patients are mental slowness, memory impairments, and attention deficits (Rijckevorsel, 2006). These cognitive impairments are also found in chemically induced animal models of TLE. The administration of pilocarpine in mice generates chronic hyperexcitability of glutamatergic DGCs increasing seizure susceptibility. These animals also exhibit impairments in spatial and discriminative memory (Kalemenev et al., 2015; Kahn et al., 2019; Smolensky et al., 2019; Park et al., 2020). To minimize the cognitive impairments that result from the progression of epilepsy, Kahn et al. (2019) expressed hM4Di in DGCs of mice with chronic epilepsy, induced by systemic administration of pilocarpine. They demonstrated that the administration of CNO reduced DG hyperexcitability, which led to the recovery of memory impairments in these epileptic mice while having no effect in green fluorescence protein-expressing controls. In a separate cohort of non-epileptic mice, they then showed that hyperexcitability induced in DGCs by hM3Dq led to spatial memory deficits comparable to epileptic mice (Kahn et al., 2019).

## Discussion

In this review, we presented examples of the application of DREADDs to epilepsy research. DREADDs has been used to manipulate a subset of cells to potentiate seizures. Additionally, groups have demonstrated DREADDs as a technique to create a seizure focus and to elucidate cellular and network mechanisms underlying seizures. Both hM3Dq and hM4Di have been used to control seizures. Several groups explored ligand dosing strategies for controlling seizures and identified important considerations for designing regimens for future experiments. Furthermore, DREADDs have been used along with other techniques to identify pathological changes in biochemistry that may lead to epilepsy. Finally, DREADDs can be used to investigate comorbidities associated with epilepsy. These experiments may help identify therapeutic targets for future treatment strategies.

### Comparison Between DREADDs and Optogenetics

There are other tools that have been used to manipulate cells to evaluate the pathophysiology of epilepsy. Optogenetics is a powerful tool for modulating neuronal activity. Cela and Sjostrom provided a thorough review of its application in epilepsy research (Cela and Sjöström, 2019). A review by Forcelli provided comparisons between optogenetics and chemogenetics in epilepsy research (Forcelli, 2017). Briefly, optogenetics allows for both the activation (e.g., channelrhodopsins) and inhibition (e.g., halorhodopsin) of neurons by light induced ion channel opening (Nagel et al., 2002; Krook-Magnuson et al., 2015; Cela et al., 2019). Optogenetics has an immediate effect on neuronal activity upon light stimulation and its stimulus has temporal resolution of milliseconds (Boyden et al., 2005; Gunaydin et al., 2010). Despite its high temporal resolution, optogenetics requires hardware implantation for light delivery (Cook et al., 2013; Krook-Magnuson et al., 2013; Paz et al., 2013). In addition, the effect of optogenetics depends on the penetration of light into the brain (Yizhar et al., 2011), which is confined to a small region around the light source. The required light stimulation when using optogenetics has posed challenges for its implementation in larger brains such as nonhuman primates due to the skull’s thickness (Herculano-Houzel, 2009; Watanabe et al., 2020). Additionally, the increased intensities necessary to activate larger regions may lead to heat generation from the fibers (Yizhar et al., 2011) which may alter the physiological properties of surrounding brain tissue (Kim and Connors, 2012). Unlike optogenetics, the actions of DREADDs are mediated either by the adenylyl cyclase signaling pathway (hM4Di) or inositol 1,4,5-trisphosphate-mediated Ca^2+^ release (hM3Dq), which results in much slower onset of their effect (Alexander et al., 2009; Forcelli, 2017; Atasoy and Sternson, 2018). The effects of DREADDs on neuronal activity start approximately 30 min after ligand administration and can last up to several hours.

An interesting solution for the limitation of optogenetics was demonstrated by Tung et al. (2018) when they combined optogenetics with chemogenetics to remove the necessity of an implanted light source. Specifically, inhibitory luminopsin, a protein resulting from the fusion of an inhibitory halorhodopsin and luciferase probe, allowed the cells that express inhibitory luminopsins to have an optogenetic-induced response by their own light-source when activated by the ligand coelenterazine. This resulted in inhibitory effects with temporal resolution comparable to external light-activated halorhodopsin, but activated by a chemical ligand. This also allows receptors in spatially distinct locations to be activated by systemic administration of a ligand. By targeting both the dentate gyrus and anterior nucleus of the thalamus, Tung et al. (2018) demonstrated the inhibitory effects of luminopsins in reducing seizures induced by pentylenetetrazol in rats.

### Challenges for Clinical Translation of DREADDs

Having presented applications of DREADDs to epilepsy research, we now consider their potential to be used in treating patients with seizure disorders. DREADDs-based therapies are promising treatment strategies to address the need for effective epilepsy treatments. The fact that the exogenous ligands of DREADDs are largely inert toward other receptors and tissues is a desirable attribute of this technique when being considered for therapeutic applications. Here, we have identified a few challenges to be met before the implementation of a DREADD construct as a form of ASD.

First, to apply one of the described DREADDs as an intervention for patients with epilepsy, the utilization of gene therapy is likely needed. Even though several gene therapies have been validated in preclinical models, the concern for untoward effects associated with the use of viral vectors to deliver gene therapies persists. Recent advancements in gene therapies are focused on minimizing potential side effects by engineering vectors with high selectivity for the targeted cells of the brain (Wang et al., 2019).

Second, since the genetic modification of neurons is likely irreversible, an optimal viral dosage to achieve the desired therapeutic effect without compromising normal brain function must be identified. The dosage of viral vectors is dependent on the number of viral copies per infected neurons, number of cells infected, and resultant level of receptor reserve in the target tissue. This requires a precise strategy to ensure that the epileptic zone is effectively transfected with minimal spread to neighboring regions (Lieb et al., 2019).

Third, decisions for current therapies that alter the genome to enter human trials are made restrictively in cases where the diseases are reported to be untreatable, or when conventional therapies are no longer effective (Lowenstein, 2008). Along with the possible safety issues and technical considerations described above, a therapy that modifies the human genome needs to take into consideration the patient’s privacy, free will, and personal identity. The legal and social implications of altering the human genome are complex (Canli, 2015).

Fourth, another challenge in applying chemogenetics to treat the diseases that affect the central nervous system is how to deliver the construct across the BBB. For this reason, the AAV carrying the DREADD viral construct has predominantly been delivered to the target brain region *via* stereotaxic surgery. The use of AAVs in neural tissue presents a safe and reliable profile with high specificity of viral vectors to infect specific subsets of cells (McCown, 2005; Bowers et al., 2011; High and Aubourg, 2011; Weinberg et al., 2013; Canli, 2015). However, the limited capacity of AAVs to cross the BBB requires intravenous delivery of different serotypes of AAV. New tools, such as drugs that transiently increase BBB permeability may overcome this problem. An alternative is the development of new AAV serotypes with higher crossing capacities, such as recombinant AAVs or human recombinant AAVs (Jackson et al., 2016; Goertsen et al., 2022). These technologies would need to be combined with more specific cellular promoters to maintain the regional specificity driven by stereotaxic injection (Hudry and Vandenberghe, 2019). Another option is intrathecal injections, either cisternal or lumbar, of the viral vectors (Hocquemiller et al., 2016).

Fifth, ligands for DREADDs activation will also need to cross the BBB. Recent publications provided evidence that CNO is reverse metabolized peripherally into clozapine and N-desmethylclozapine. Instead of CNO, these metabolites cross the BBB and possess higher binding affinity to muscarinic DREADDs than CNO (Nawaratne et al., 2008; Hellman et al., 2016; Gomez et al., 2017; Manvich et al., 2018). A potential alternative to requiring ligand delivery was made by Lieb et al. (2018) when they engineered an antiepileptic autoregulatory chemogenetic therapy. Their technique consisted of genetically modifying a glutamate-gated chloride channel gene found in round worms (*Caenorhabditis elegans*) to have higher sensitivity to glutamate. Pilocarpine-induced epileptic mice that had these enhanced glutamate-gated chloride channels delivered into cortical pyramidal neurons showed attenuated acute seizures and a progressive decrease in the number and frequency of seizures in the chronic period of epilepsy (Lieb et al., 2018). Most interestingly, despite activity of enhanced glutamate-gated chloride channels being regulated by endogenous glutamate, expression of these modified channels in the brain does not affect normal brain function. This is most likely due to the efficient clearance of glutamate from extrasynaptic spaces by excitatory amino acid transporters (Tzingounis and Wadiche, 2007; Lieb et al., 2018). These results increase the likelihood of finding appropriate ligands for DREADDs as a treatment modality.

Finally, another important consideration when applying DREADDs to treating patients with epilepsy is the longevity of the treatment. The substantial effect and relatively long duration of 8 h after administration of ligand currently make DREADDs an ideal rescue treatment upon administration of the ligand. Long-term dosing of the ligand as a chronic treatment requires further development and consistency across researchers in designing experiments. It is unknown whether the tolerance effect presented by Goossens et al. (2021) above could have been avoided by changing the dosing schedule or increasing the number of expressed receptors since it was originally hypothesized that receptor reserve would protect against tolerance. Furthermore, tolerance effects have not been evaluated when targeting interneurons with DREADDs. An alternative would be to develop a DREADD construct that was resistant to desensitization. Since DREADDs require the same cellular machinery that GPCRs use to mediate their effect, they are also subject to phosphorylation-dependent effects such as desensitization and arrestin-mediated receptor degradation (Yu et al., 1993). These cellular mechanisms can lead to the tolerance effects described above. The neuronal effects of DREADDs are largely thought to be mediated by GPCR mechanisms (Armbruster et al., 2007) so biased receptors may still mediate the intended effect without being desensitized. To evaluate adverse effects that may be associated with the use of cholinesterase inhibitors in Alzheimer’s disease, Bradley et al. (2020) developed a phosphorylation-deficient DREADD. Knocking in this DREADD in mice resulted in expression in cells instead of the wild-type receptor. They demonstrated that treatment of these mice with CNO resulted in TLE-like seizures that were comparable to seizures induced by pilocarpine (Bradley et al., 2020). Although they did not show long-term effects of their phosphorylation-deficient DREADD, this DREADD construct or a phosphorylation deficient version of hM3Dq or hM4Di may be resistant to phosphorylation-dependent receptor degradation, and of potential use in chronic treatment for epilepsy.

Prior to clinical use, DREADDs must be demonstrated safe and efficacious in non-human primates. As a first step, researchers investigated whether the use of rodent-optimized viruses can transduce and drive the required levels of DREADD expression in these primates. To evaluate this, Galvan et al. (2019) performed an ultrastructural analysis of DREADD location in non-human primate and mouse neurons to verify whether differences across species might impact the subcellular location and plasma membrane expression of DREADDs. They reasoned that the neurons that express DREADDs in locations other than the plasma membrane would be unable to modulate neural activity. Using the same virus construct to express hM4Di fused to the mCherry fluorescent reporter protein, they showed that individual DREADDs were expressed mainly in the plasma membrane of mice. However, in non-human primate brain tissue, the receptors were found distributed in the intracellular space, where they are not able to perform their modulatory action (Galvan et al., 2019).

## Conclusion

In summary, DREADDs have proven to be powerful tools for improving our understanding of the pathophysiology of epilepsy. DREADDs have the potential to become new treatment modalities for patients suffering from this disease. Further investigations are needed in order to apply the laboratory findings to improve treatment outcomes for these patients.

## Author Contributions

All authors contributed equally to the planning, research, writing, review, and development of this work. All authors contributed to the article and approved the submitted version.

## Conflict of Interest

The authors declare that the research was conducted in the absence of any commercial or financial relationships that could be construed as a potential conflict of interest.

## Publisher’s Note

All claims expressed in this article are solely those of the authors and do not necessarily represent those of their affiliated organizations, or those of the publisher, the editors and the reviewers. Any product that may be evaluated in this article, or claim that may be made by its manufacturer, is not guaranteed or endorsed by the publisher.
